# Atopic dermatitis and lymphoma risk: a systematic review and meta-analysis

**DOI:** 10.3389/fonc.2025.1580550

**Published:** 2025-04-14

**Authors:** Yingjie Tian, Yujin Li, Yang Chen, Guoxing Yuan, Bowen Peng, Liang Su, Jie Wu

**Affiliations:** ^1^ Department of Hematology, Guang’anmen Hospital, China Academy of Chinese Medical Sciences, Beijing, China; ^2^ Graduate School, Beijing University of Chinese Medicine, Beijing, China; ^3^ Department of Hematology, The First People’s Hospital of Yunnan Province, The Affiliated Hospital of Kunming University of Science and Technology, Kunming, Yunnan, China; ^4^ Yunnan Provincial Clinical Medical Center for Blood Diseases and Thrombosis Prevention and Treatment, The First People’s Hospital of Yunnan Province, Kunming, Yunnan, China; ^5^ Yunnan Provincial Key Laboratory of Innovative Application of Characteristic Chinese Materia Medica, The First People’s Hospital of Yunnan Province, Kunming, Yunnan, China; ^6^ Department of Pathology, The First People’s Hospital of Yunnan Province, Kunming, Yunnan, China; ^7^ Department of Pathology, The Affiliated Hospital of Kunming University of Science and Technology, Kunming, Yunnan, China

**Keywords:** atopic dermatitis, lymphoma, T-cell lymphoma, systematic review, meta-analysis

## Abstract

**Background:**

The relationship between atopic dermatitis (AD) and lymphoma risk remains debate. This study systematically evaluates lymphoma risk in AD patients compared to non-AD individuals.

**Methods:**

A systematic search of PubMed, Embase, and the Cochrane Library (up to August 11, 2024) identified observational studies reporting lymphoma risk estimates for AD patients. Pooled odds ratios (OR) or relative risks (RR) with 95% CIs were calculated using a random-effects model (PROSPERO ID: CRD42024577019).

**Results:**

Of 2,366 articles were screened, 13 studies met the inclusion criteria. AD was significantly associated with elevated lymphoma risk (OR = 2.56, 95% CI: 1.75–3.74, P < 0.001; RR = 1.23, 95% CI: 1.15–1.31, P < 0.001). The risk increased with AD severity, with severe cases showing the highest effect size (RR = 2.63; 95% CI: 1.94–3.58, P < 0.001; OR = 2.60; 95% CI: 1.71–3.96, P < 0.001). Subgroup analyses revealed high risks for Hodgkin lymphoma (HL) (RR = 1.54, 95% CI: 1.35–1.75, P < 0.001) and non-Hodgkin lymphoma (RR = 1.15, 95% CI: 1.04–1.28, P = 0.006). Notably, T-cell lymphoma (TCL) showed the highest risk (OR = 4.25; 95% CI: 1.94–9.33, P < 0.001). whereas no significant association was observed for B-cell lymphoma (OR = 1.07; 95% CI: 0.95–1.20, P = 0.271).

**Conclusion:**

AD is significantly association with increased lymphoma risk, particularly HL, NHL and TCL. AD severity may amplify this risk. Future research is warranted to explore underlying mechanisms and address limitations in the current evidence.

**Systematic review registration:**

https://www.crd.york.ac.uk/PROSPERO/, identifier CRD42024577019.

## 1 Introduction

Atopic dermatitis (AD) is a prevalent chronic inflammatory skin disease with rising global incidence, affecting approximately 15–20% of children and 10% of adults. Recognized as the most burdensome non-fatal dermatological condition worldwide, AD significantly contributes to the global disease burden (1–5). Patients with AD often endure severe pruritus and recurrent eczema, leading to insomnia and psychological comorbidities such as depression and anxiety, which markedly diminish their quality of life and that of their families (6–9). The pathogenesis of AD involves a complex interplay between genetic and environmental factors, with persistent immune activation, skin barrier dysfunction, and microbiome dysbiosis identified as key mechanisms (1, 2, 10, 11). These interconnected processes can drive systemic chronic inflammation and immune dysregulation. Furthermore, therapies such as dupilumab and immunosuppressive agents, including topical corticosteroids (TCSs) and calcineurin inhibitors (TCIs), have been associated with an increased risk of malignancies, particularly lymphomas (12–14).

Lymphoma, a heterogeneous group of malignancies originating in the lymphatic system, is characterized by significant etiological diversity. Common risk factors include immune system abnormalities, viral infections, air pollution, and occupational exposures, with immune dysfunction playing a central role in lymphoma pathogenesis (15, 16). As a prototypical chronic immune-stimulating condition, AD is associated with persistent immune activation (17). Studies have reported a heightened risk of lymphoma in AD patients, particularly those with severe disease or prolonged use of high-potency corticosteroids. Additionally, some research has proposed a potential link between childhood AD and the subsequent development of non-Hodgkin lymphoma (NHL) (18–23). However, the evidence remains inconsistent. While some studies suggest that the hyperactive immune state in AD may enhance immunosurveillance, potentially reducing cancer incidence (12, 24), others have failed to establish a definitive causal relationship between AD and lymphoma or other malignancies (25). Further complicating this relationship is the significant clinical overlap between AD and cutaneous T-cell lymphoma (CTCL), which poses diagnostic challenges and risks of misclassification (26, 27). Given these discrepancies and the substantial global burden of AD, this study aims to systematically evaluate the association between AD and lymphoma risk through a systematic review and meta-analysis of observational studies.

## 2 Methods

This systematic review and meta-analysis followed the Preferred Reporting Items for Systematic Reviews and Meta-Analyses (PRISMA) guidelines (PROSPERO ID: CRD42024577019) (28). Ethical approval was not required, as the study exclusively utilized data from previously published sources.

### 2.1 Data sources and searches

A comprehensive search was conducted in PubMed, EMBASE, and The Cochrane Library for articles published from inception to August 11, 2024. Only studies published in English were included. To ensure thorough identification of relevant studies, a combination of MeSH terms and free-text keywords was employed. Key search terms included “dermatitis, atopic,” “atopic dermatitis,” “eczema,” “lymphoma,” “lymphoproliferative neoplasm,” and “chronic lymphatic leukemia.” Reference lists of included studies were also screened for additional relevant articles. No restrictions were imposed on population, ethnicity, geographic region, age, or study period. The detailed search strategy is provided in **Supplementary Appendix 1**.

### 2.2 Study selection

Two reviewers (LS and Y-j T) independently screened titles and abstracts to identify potentially eligible studies. Full-text articles passing the initial screening were further evaluated for eligibility. Reasons for exclusion were systematically recorded. Discrepancies during the screening or data extraction process were resolved by consensus with a third reviewer (JW). All reviewers underwent standardized training to ensure consistency before initiating the formal review. Inclusion criteria were as follows: 1) Studies including at least one group of patients with AD; 2) A comparison group consisting of non-AD individuals or the general population; 3) Investigation of lymphoma incidence rates. Exclusion criteria were as follows: 1) Studies lacking sufficient data for analysis; 2) Duplicate studies based on the same patient population; only the most recent publication was retained.

### 2.3 Data extraction and quality assessment

Data extracted from eligible studies included: first author, publication year, country, study period, age, sample size, lymphoma classification, and effect estimates (e.g., odds ratios [ORs] and relative risks [RRs]). Study quality was assessed using the Newcastle-Ottawa Scale (NOS), a validated tool for evaluating the risk of bias in cohort and case-control studies. The NOS evaluates three domains: selection of study groups (maximum 4 points), comparability (maximum 2 points), and outcome or exposure assessment (maximum 3 points). Total scores ranged from 0 to 9, with scores ≥8 considered high quality, 5–7 moderate quality, and <5 low quality (29, 30). Data extraction and quality assessment were independently performed by two reviewers (LS and Y-j T), with discrepancies resolved through discussion with a third reviewer (JW). Detailed scoring results are presented in **Table 1**.

**Table 1:** Baseline characteristics of the included studies.

Study	Location	Age	Inclusion Period	No. of patients/controls	Lymphoma type	Main results (95%CI)	Quality
Arellano et al., 2007 (34)	USA	case: 47.8 (0-61+) controls: 30.4 (0-61+)	July 1995 to January 2005	294 cases of lymphoma/1176	HL、NHL	OR=2.40(1.50, 3.80)	8
Arellano et al., 2009 (35)	UK	case: 49.65(0-61+) controls: 48.01 (0-61+)	January 1, 1992 to March 23, 2006	2738 case of lymphoma/10,949	HL、NHL	OR=1.83(1.41, 2.36)	8
Engels et al., 2016 (36)	USA	case: 77.28(65-85+) controls: 76.77(65-85+)	July 1, 1992 to July 1, 2009	52,691 cases of NHL/200,000	NHL	OR=1.29(1.18, 1.41)	7
Joshi et al., 2023 (37)	USA	55.0 ± 12.7	NR	174 cases of CTCL/696	MF	OR=9.48(4.86, 18.51)	8
Jung et al., 2023 (38)	Korean	exposed: 19.15(<10-≥60) controls: 19.15(<10-≥60)	2003 to 2019	254, 644 patients with AD/254, 644 subjects without AD	NK/TCL	RR=2.83(1.12, 7.19)	8
Kaul et al., 2019 (39)	USA	61.36(18-110)	January 1, 2013 and January 1, 2019	580 patients with MF/4,943,869 patients in the general population; 10,382 patients with AD	MF	OR=19.70(13.00, 29.90)	4
Mansfield et al., 2020 (40)(A)	England	exposed: 45.55(18-65+) controls: 44.45(18-65+)	January 2, 1998, to March 31, 2016	471,970 patients with AD/2, 239, 775 subjects without AD	NHL、HL	RR=1.22(1.13, 1.32)	8
Mansfield et al., 2020 (40)(B)	Denmark	exposed:17.57(<18-≥65) controls: 17.38(<18-≥65)	January 1, 1982, to June 30, 2016	44,945 patients with AD/445, 673 subjects without AD	NHL、HL	RR=1.32(0.95, 1.84)	8
Morales et al., 2003 (41)	European^①^	case: 56.03(35-69) controls: 54.11(35-69)	1995 to 1998	76 patients with MF/2904	MF	OR=1.60(0.80, 3.00)	7
Pierog et al., 2024^③^ (42)	MCAR^②^	NR	2001 to 2023	1,056,813 patients with AD/1,056,813	CTCL	OR=1.64(1.43, 1.88)	8
Powers et al., 2024 (18)	USA	case: 56(39-67) controls: 56(39-67)	2018 to 2024	6425 patients with AD/25,700	BCL、NCTCL	OR=1.43(1.12, 1.84)	7
Ruff et al., 2017 (43)	Denmark	42.2(≥18)	January 1, 1997 to December 31, 2012	8,112 patients with AD/40,560	lymphoma	OR=1.86(1.43, 2.40)	6
Tuyp et al., 1987 (44)	NR	case: 51(11-82) controls: 51(11-82)	NR	53 patients with MF/53	MF	OR=2.04(0.18, 23.12)	6
Wan et al., 2023 (19)(A)	UK	case: 4(<18) controls: 4-9(<18)	1994 to February 2015	409,431 children with AD/1,809,029	HL、NHL	RR=1.44(1.11, 1.85)	9
Wan et al., 2023 (19)(B)	UK	case: 47(≥18) controls: 45-47(≥18)	1994 to February 2015	625,083 adults with AD/2,678,888	HL、NHL	RR=1.23(1.15, 1.31)	9

①European(Denmark, Sweden, France, Germany, Italy, Spain); ②This study includes data from multiple countries and regions (MCAR), including the United States, Europe, Latin America, and the Asia-Pacific region; ③This is a retrospective cohort study reporting odds ratios (ORs), and we included it in our analysis as a case-control study.

HL, Hodgkin-lymphoma; NHL, non-Hodgkin lymphoma; CLL/SLL, chronic lymphocytic leukemia/small lymphocytic lymphoma; DLBCL, diffuse large B-cell lymphoma; FL, follicular lymphoma; MZL, marginal zone lymphoma; TCL, T-cell lymphoma; MF, Mycosis fungoides; NK/TCL, NK/T cell lymphoma; CTL, Cutaneous T-cell lymphomas; BCL, B-cell lymphomas; NTCL, Non-CTCL T-cell lymphoma.

### 2.4 Statistical analysis

Risk estimates were expressed as RRs or ORs with 95% CIs, with hazard ratios (HRs) considered equivalent to RRs (31). For studies reporting stratified results, a fixed-effect meta-analysis was applied to derive overall risk estimates (32). To account for potential clinical heterogeneity and enhance robustness, the DerSimonian-Laird random-effects model was used for meta-analysis (33). Heterogeneity among studies was assessed using Cochran’s Q test and the I² statistic, with I² ≥50% indicating moderate to substantial heterogeneity. Sensitivity analyses were conducted sequentially excluding individual studies to evaluate result robustness. Subgroup analyses were performed based on lymphoma classification and AD severity. For datasets with more than 10 studies, publication bias was evaluated visually using funnel plots and quantitatively using Begg’s and Egger’s tests (33). All statistical analyses were performed using Stata version 17.0, with P < 0.05 considered statistically significant.

## 3 Results

### 3.1 Search results

**Figure 1:**
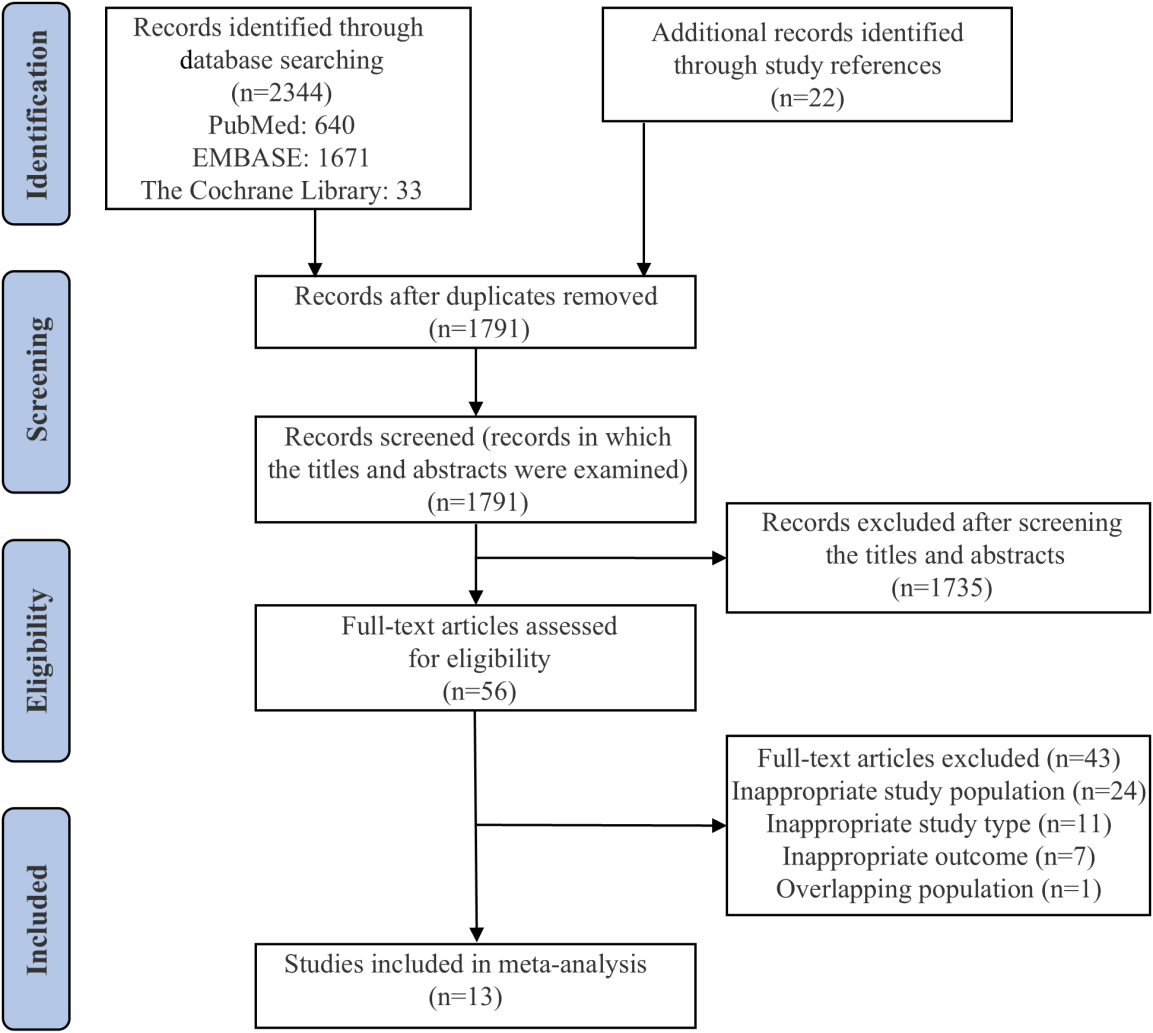
Flow diagram for the study selection process.

A total of 2,344 references were identified through database searches, supplemented by 22 additional references from citation reviews. After screening titles and abstracts and removing duplicates, 2,310 articles were excluded. The full texts of 56 articles were assessed for eligibility, and 13 studies ultimately met the inclusion criteria (18, 19, 34–44). The detailed literature selection process is depicted in **Figure 1** and elaborated in **Supplementary Appendix 2**.

### 3.2 Study characteristics and quality assessment

The characteristics of the included studies are summarized in **Table 1**. This systematic review and meta-analysis incorporated nine case-control studies (18, 34–37, 39, 41, 43, 44) and four cohort studies (19, 38, 40, 42). The case-control studies involved 71,143 participants, conducted across the United States (five studies) (18, 34, 36, 37, 39), the United Kingdom (one study) (35), and Europe(two studies) (41, 43). Study periods ranged from 1992 to 2024, with participants’ mean age spanning 30 to 78 years, most averaging around 50 years. One study exclusively included participants aged 65 years or older (36). Lymphoma subtypes varied across studies: two studies examined both HL and NHL (34, 35); one focused solely on NHL (36); one investigated B-cell lymphomas(BCL) and non-cutaneous T-cell lymphomas(NCTCL) (18); four examined mycosis fungoides(MF) exclusively (37, 39, 41, 44); and one did not specify lymphoma subtypes (43).

The four cohort studies included six cohorts with a combined total of 2,862,886 participants from the United Kingdom (three cohorts) (19, 40), Denmark (one cohort) (40), South Korea (one cohort) (38), and a multinational retrospective cohort study covering the United Kingdom, Europe, Latin America, and the Asia-Pacific region (42). Study periods spanned from 1982 to 2023, with participants ages 4 to 50 years. Two cohort studies focused exclusively on adults (19, 40), while one included only children (19). Among these, four studies reported both HL and NHL (19, 40); one exclusively studied on CTCL (42); and one examined only T-cell lymphomas(TCL) (38). Study quality, assessed using the Newcastle-Ottawa Scale (NOS), indicated high average scores: cohort studies averaged 8.3 (range: 8–9), and case-control studies averaged 7.1 (range: 6–8). Detailed NOS evaluations are presented in **Table 1**.

### 3.3 Risk of lymphoma

**Figure 2:**
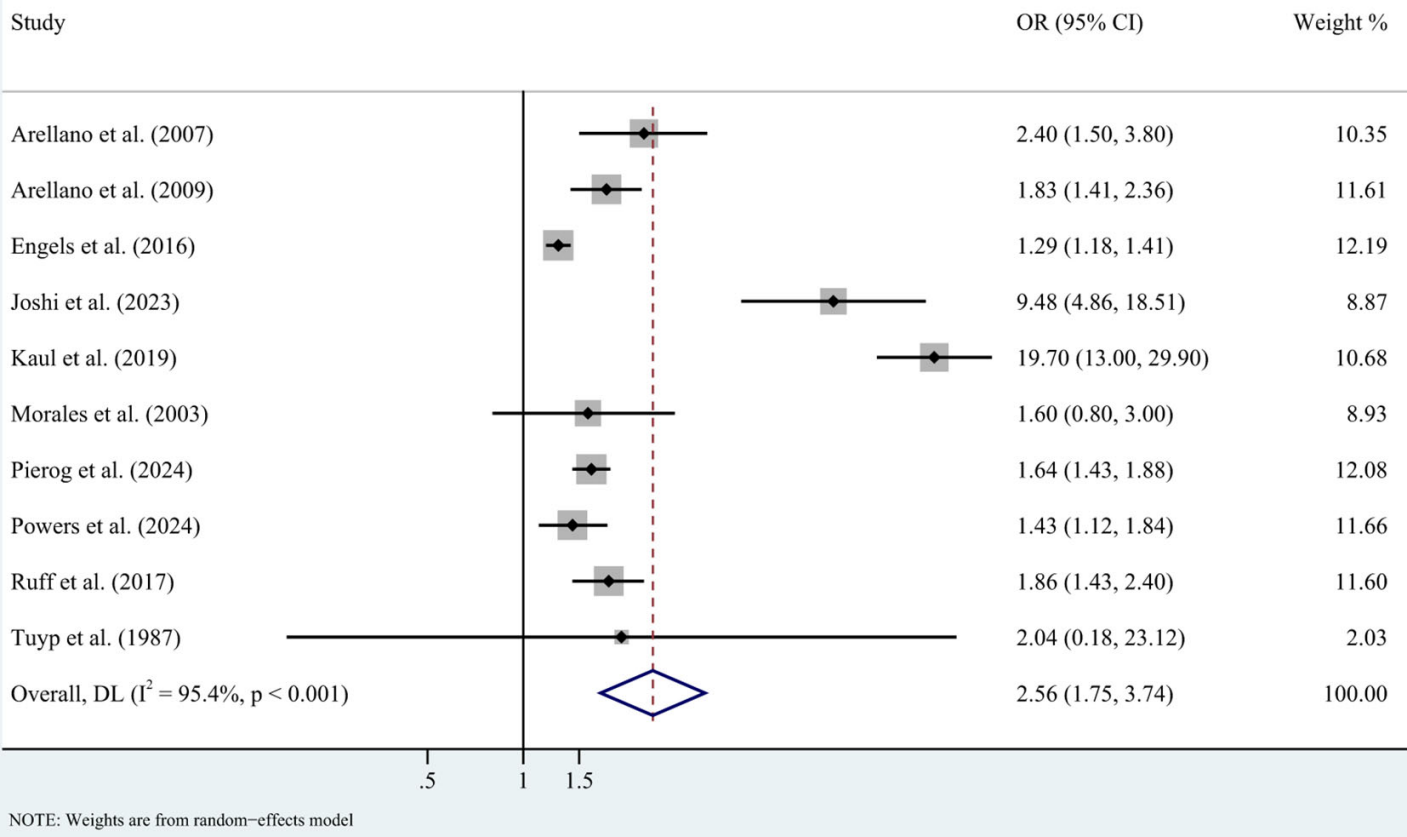
Forest plot for meta-analysis on the association between atopic dermatitis and lymphoma risk in case-control studies (odds ratios). CI, confidence interval; DL, DerSimonian-Laird estimate; I^2^, inconsistency.

**Figure 3:**
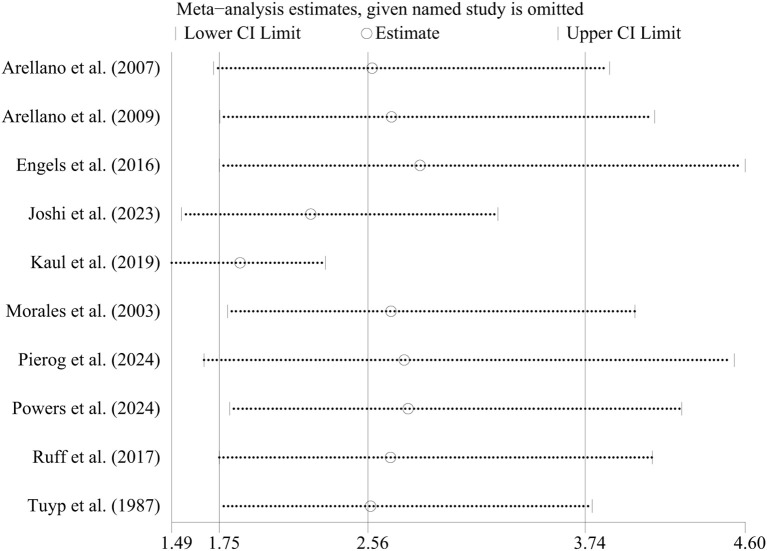
Sensitivity analysis of the association between atopic dermatitis and lymphoma risk in case-control studies. CI, confidence interval.

**Figure 4:**
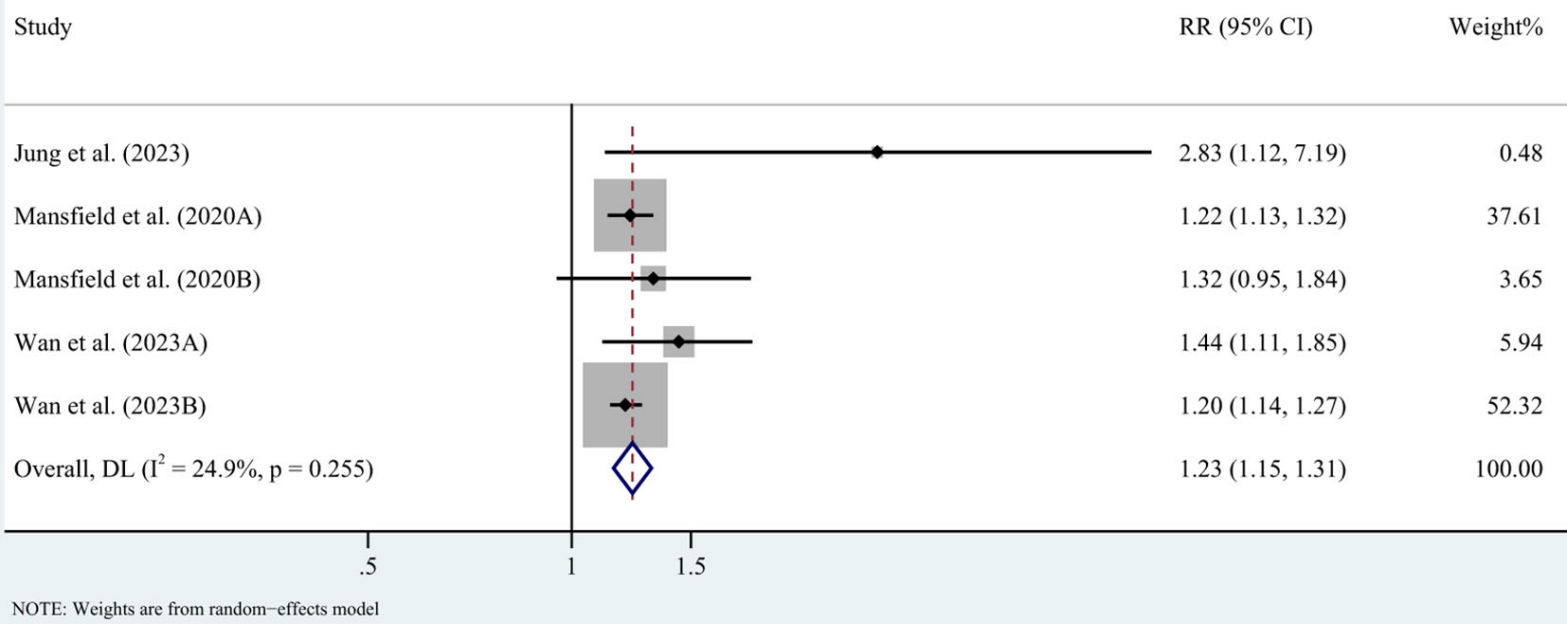
Forest plot for meta-analysis on the association between atopic dermatitis and lymphoma risk in cohort studies (relative risks). CI, confidence interval; DL, DerSimonian-Laird estimate; I^2^, inconsistency.

**Figure 5:**
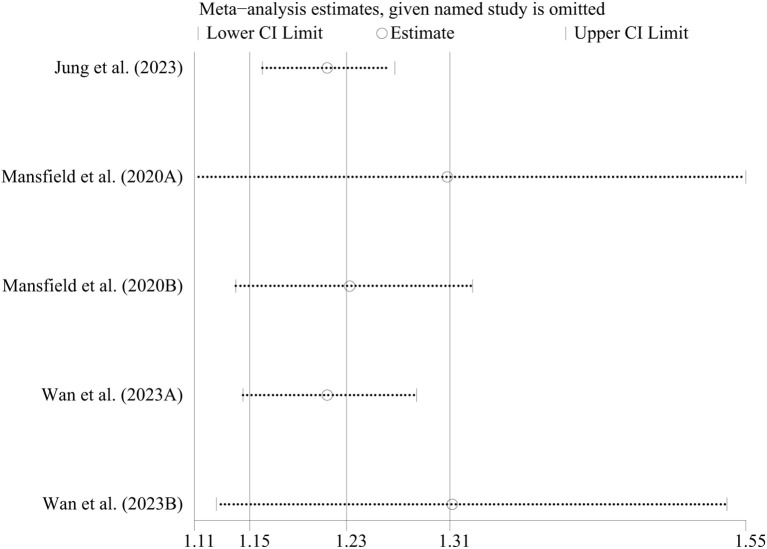
Sensitivity analysis of the association between atopic dermatitis and lymphoma risk in cohort-control studies. CI, confidence interval.

In case-control studies, the meta-analysis revealed a significant association between AD and lymphoma, with a pooled OR of 2.56 (95% CI: 1.75–3.74, P < 0.001). Substantial heterogeneity was noted (I² = 95.4%, P < 0.001) (**Figure 2**). Sensitivity analyses confirmed the robustness of these findings, as the exclusion individual studies did not significantly alter the results (**Figure 3**). In cohort studies, AD was also significantly associated with an increased risk of lymphoma, with a pooled RR of 1.23 (95% CI: 1.15–1.31, P < 0.001). Heterogeneity was low and not statistically significant (I² = 24.9%, P = 0.255) (**Figure 4**). Sensitivity analyses supported the stability of these results, with no material impact observed when individual studies were excluded (**Figure 5**).

### 3.4 Subgroup analysis according to AD severity

For mild AD, the pooled RR was 1.09 (95% CI: 1.02–1.16, P = 0.012; I² = 0%, P = 0.422). For moderate AD, the pooled RR increased to 1.29 (95% CI: 1.21–1.39, P < 0.001; I² = 0%, P = 0.484). Severe AD showed a markedly higher lymphoma risk, with a pooled RR of 2.63 (95% CI: 1.94–3.58, P < 0.001; I² = 64.2%, P = 0.061) (**Supplementary Figure 1**). Case-control studies similarly identified significant associations between severe AD and lymphoma risk, yielding a pooled OR of 2.60 (95% CI: 1.71–3.96, P < 0.001). No significant heterogeneity was observed (I² = 0%, P = 0.427) (**Supplementary Figure 2**). These findings indicate a positive correlation between AD severity and lymphoma risk, with severe AD presenting the largest effect size.

### 3.5 Subgroup analysis according to lymphoma classification

Lymphomas, a diverse group of malignancies, are broadly categorized into HL and NHL based on the presence of Reed-Sternberg cells. Subgroup analysis revealed significant associations between AD and risks of both HL and NHL. The pooled RR for HL was 1.54 (95% CI: 1.35–1.75, P < 0.001, I² = 0%, P = 0.822), while for NHL, the pooled RR was 1.15 (95% CI: 1.04–1.28, P = 0.006, I² = 44.2%, P = 0.127) (**Supplementary Figure 3**).

When lymphomas were further classified by cell origin into B-cell and T-cell subtypes, no significant association was observed between AD and BCL, the pooled OR was 1.07 (95% CI: 0.95–1.20, P = 0.271); no evidence of heterogeneity (I² = 0%, P=0.532). In contrast, a strong association was observed between AD and TCL, the pooled OR was 4.25 (95% CI: 1.94–9.33, P<0.001), although substantial heterogeneity was noted (I² = 96.4%, P<0.001) (**Supplementary Figure 4**). These results suggest that the risk of lymphoma associated with AD varies by subtype, with TCL exhibiting the strongest association.

### 3.6 Publication bias

**Figure 6:**
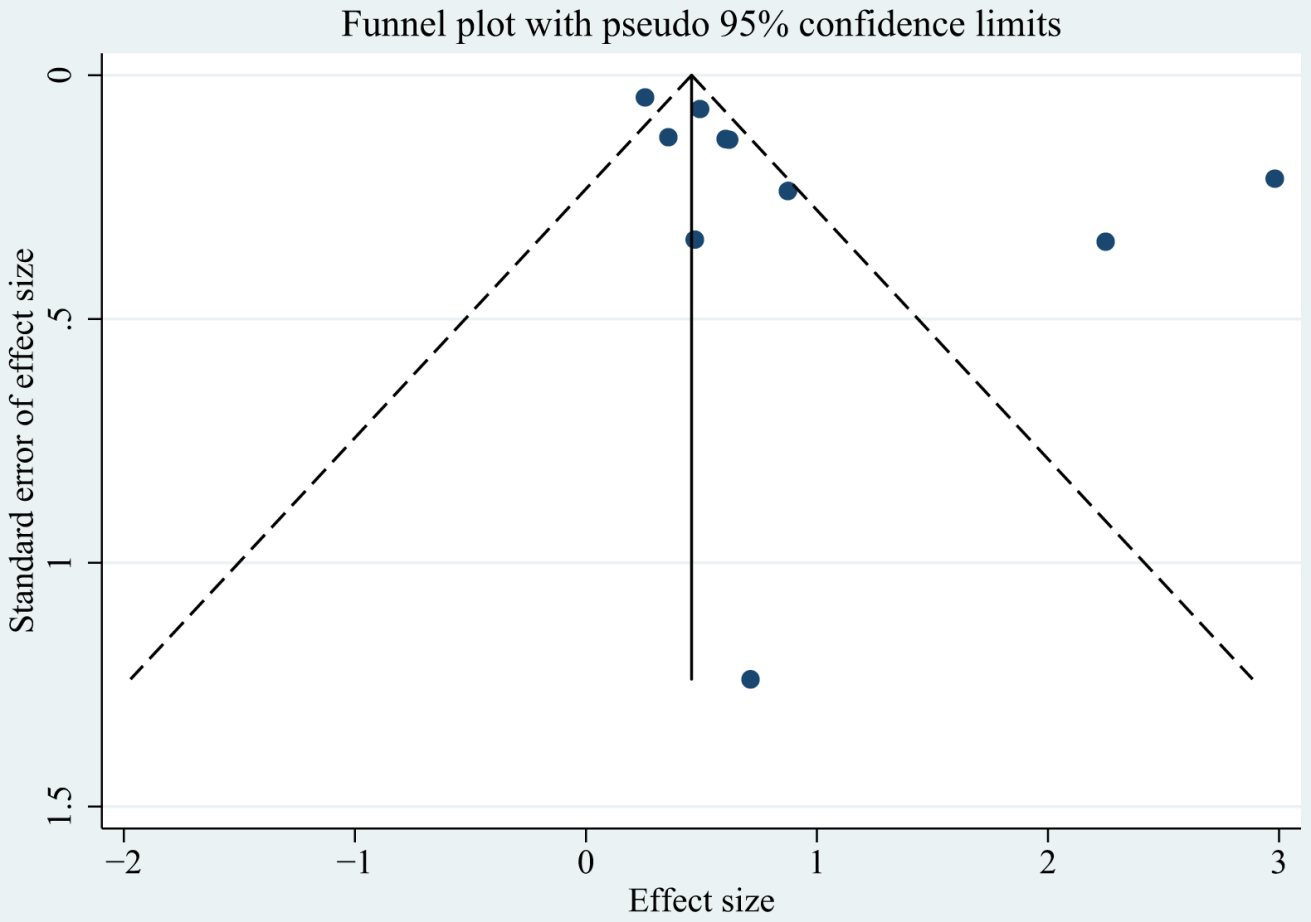
The funnel plots of the association of atopic dermatitis and lymphoma risk.

Among 10 case-control studies, publication bias was assessed using funnel plots (**Figure 6**), Egger’s test, and Begg’s test. While the funnel plot showed slight asymmetry, neither Egger’s test (P = 0.063) (**Supplementary Figure 5**) nor Begg’s test (P = 0.152) (**Supplementary Figure 6**) indicated significant publication bias.

## 4 Discussion

Our findings demonstrate a statistically significant association between AD and increased risk of lymphoma. Particularly for HL, NHL, and TCL. Additionally, lymphoma risk was positively correlated with AD severity, with severe AD exhibiting the highest effect size.

The potential link between AD and lymphoma risk was first reported in 1989, when a case report described the progression of AD in a pediatric patient to fatal CTCL (45). Our subgroup analysis further highlights the significant association between AD and an elevated risk of TCL. This finding may partially stem from the clinical overlap between MF, the most common subtype of CTCL, and AD, which often complicates diagnosis and increases the likelihood of misclassification. MF accounts for approximately half of all CTCL cases, emphasizing the need for heightened clinical vigilance (46–49). Notably, a case-control study that explicitly excluded CTCL still observed an elevated risk of TCL in AD patients (18), suggesting that this association cannot be solely attributed to diagnostic misclassification. Lymphomas are a heterogeneous group of malignancies typically categorized as HL or NHL, or by cell origin as TCL or BCL (50–52). Our analysis identified significant associations between AD and increased risks of HL, NHL, and TCL. However, no significant association was found between AD and BCL, potentially due to the limited number of studies (only two) investigating BCL. Further research is required to clarify this relationship (53, 54).

A previous meta-analysis conducted by Laureline Legendre et al. (2015) reviewed 22 studies, including cohort and case-control designs, to evaluate the association between AD and lymphoma risk (55). Their analysis found an increased lymphoma risk in cohort studies but no significant association in case-control studies. In contrast, our meta-analysis demonstrated elevated lymphoma risks in both study types. This discrepancy may arise from our stricter inclusion criteria, which excluded patients with eczema. While eczema and AD share clinical features, AD is a distinct clinical entity with unique characteristics (56). By focusing exclusively on AD, our analysis provides a more precise framework for exploring its association with lymphoma risk. Additionally, subtype-specific analyses in our study offer novel insights into the heterogeneity of lymphoma risk in AD populations.

Several plausible mechanisms may explain the observed association between AD and lymphoma risk. One leading hypothesis is the antigen stimulation theory, which posits that chronic immune activation in inflammatory diseases, such as AD, can drive oncogenesis. Persistent immune stimulation may result in heightened immune cell proliferation, increasing the likelihood of random oncogenic mutations and ultimately contributing to lymphoma development (20, 57, 58).

Additionally, skin barrier dysfunction, a hallmark of AD, could further amplify lymphoma risk. Mutations in the filaggrin gene (FLG) compromise skin barrier integrity, leading to increased transepidermal water loss, microbial dysbiosis, and colonization by *Staphylococcus aureus* (*S. aureus*) (1, 2, 10, 59–61). This bacterium produces enterotoxins that activate STAT3 signaling in malignant T cells, promoting immune dysregulation and malignant cell survival. Mouse models have demonstrated that bacterial triggers can exacerbate disease progression in genetically susceptible backgrounds of CTCL (62–64). Moreover, skin barrier disruption heightens susceptibility to antigens and pathogens, potentially compounding the risk of immune dysregulation and lymphoma (57, 59).

Thirdly, a shared signaling pathway may underlie the observed association between AD and lymphoma risk. The positive correlation between AD severity and lymphoma risk observed in our findings suggests that heightened immune activation and the use of high-potency immunosuppressants may play a crucial role. Functional germline mutations in STAT6, implicated in severe allergic conditions such as primary atopic diseases (PAD), have been identified as key drivers of this association (65–68). STAT6 mutations, frequently detected in follicular lymphoma and other BCLs, point to a potential overlap in the IL-4/JAK/STAT6 signaling pathway involved in both AD and lymphoma pathogenesis (69–71). Aberrant activation of STAT6 may predispose individuals with severe AD to lymphoma by promoting a pro-oncogenic immune environment. Further exploration of this pathway is essential to uncover potential therapeutic targets that could mitigate lymphoma risk in AD patients.

Fourthly, the use of immunosuppressants may also contribute to the increased lymphoma risk. Topical immunomodulatory agents, such as topical corticosteroids (TCSs) and calcineurin inhibitors (TCIs), are fundamental to effective disease management (72, 73). However, prolonged and systemic use of these agents has been associated with an increased lymphoma risk, possibly through mechanisms like Epstein-Barr virus (EBV) reactivation (20, 55). High-potency TCS and TCI use, particularly in combination, have been linked to this heightened risk (35, 55). Moreover, the chronic nature of AD necessitates long-term follow-up, which may inadvertently lead to increased detection of CTCL, contributing to the observed association between AD and lymphoma.

There are several limitations to consider. First, although Egger’s test and Begg’s test did not show significant publication bias, focusing on peer-reviewed literature meant that grey literature was excluded. Therefore, potential publication bias could not be completely excluded. Second, although some studies suggest variations in the association between AD and lymphoma risk among different ethnic groups (15, 74, 75), the lack of available data prevented the study from conducting stratified analyses based on ethnicity or geographic region. Future research should prioritize robust stratification to uncover population-specific nuances in this association. Third, the use of topical immunomodulatory agents, such as topical corticosteroids (TCSs) and calcineurin inhibitors (TCIs), may influence the association between AD and lymphoma risk. In our study, only three referenced studies reported on treatment scenarios (34, 35, 44). In addition, there may be an age difference in the risk of developing lymphoma in patients with AD. Furthermore, there may also be a genetic predisposition in NHL, and individuals with a family history of NHL are at a higher risk of developing lymphoma (15, 76). However, the limited data included in the study prevented stratified analyses of these factors. Future research should prioritize well-designed observational studies that address these gaps, incorporate robust stratification by age and genetic predisposition, and explore the specific impact of AD treatments on lymphoma risk. Such efforts are critical to validating and refining our understanding of the mechanisms underlying this association and uncovering population-specific nuances.

## 5 Conclusions

Our findings reveal a significant association between AD and increased risks of both HL and NHL, with the strongest correlation observed for TCL. Furthermore, lymphoma risk appears to be positively correlated with AD severity, as patients with severe AD exhibit the highest effect size. These results underscore the importance of implementing early prevention strategies and ensuring vigilant lymphoma surveillance, particularly for individuals with severe AD. However, the limitations of this study highlight the need for future research to address key confounding factors and examine lymphoma risk across different subtypes. Such efforts are crucial to deepening our understanding of the mechanisms linking AD and lymphoma, thereby validating and expanding upon the current findings.

## Data Availability

The original contributions presented in the study are included in the article/**Supplementary Material**. Further inquiries can be directed to the corresponding authors.

## References

[1] Ständer S . Atopic dermatitis. N Engl J Med. (2021) 384:1136–43. doi: 10.1056/NEJMra2023911 33761208

[2] Traidl-Hoffmann C Afghani J Akdis CA Akdis M Aydin H Bärenfaller K . Navigating the evolving landscape of atopic dermatitis: challenges and future opportunities: the 4th davos declaration. Allergy. (2024) 79:2605–24. doi: 10.1111/all.16247 39099205

[3] Laughter MR Maymone MBC Mashayekhi S Arents BWM Karimkhani C Langan SM . The global burden of atopic dermatitis: lessons from the global burden of disease study 1990-2017. Br J Dermatol. (2021) 184:304–9. doi: 10.1111/bjd.19580 33006135

[4] Nutten S . Atopic dermatitis: global epidemiology and risk factors. Ann Nutr Metab. (2015) 66 Suppl 1:8–16. doi: 10.1159/000370220 25925336

[5] Lee HH Patel KR Singam V Rastogi S Silverberg JI . A systematic review and meta-analysis of the prevalence and phenotype of adult-onset atopic dermatitis. J Am Acad Dermatol. (2019) 80:1526–32.e7. doi: 10.1016/j.jaad.2018.05.1241 29864464

[6] Chovatiya R . Atopic dermatitis (Eczema). Jama. (2023) 329:268. doi: 10.1001/jama.2022.21457 36648466 PMC10190158

[7] Yew YW Thyssen JP Silverberg JI . A systematic review and meta-analysis of the regional and age-related differences in atopic dermatitis clinical characteristics. J Am Acad Dermatol. (2019) 80:390–401. doi: 10.1016/j.jaad.2018.09.035 30287309

[8] Lei D Yousaf M Janmohamed SR Vakharia PP Chopra R Chavda R . Validation of four single-item patient-reported assessments of sleep in adult atopic dermatitis patients. Ann Allergy Asthma Immunol. (2020) 124:261–6. doi: 10.1016/j.anai.2019.12.002 31830585

[9] Patel KR Immaneni S Singam V Rastogi S Silverberg JI . Association between atopic dermatitis, depression, and suicidal ideation: A systematic review and meta-analysis. J Am Acad Dermatol. (2019) 80:402–10. doi: 10.1016/j.jaad.2018.08.063 30365995

[10] Narla S Silverberg JI . The role of environmental exposures in atopic dermatitis. Curr Allergy Asthma Rep. (2020) 20:74. doi: 10.1007/s11882-020-00971-z 33047271

[11] Mohammad S Karim MR Iqbal S Lee JH Mathiyalagan R Kim YJ . Atopic dermatitis: pathophysiology, microbiota, and metabolome - a comprehensive review. Microbiol Res. (2024) 281:127595. doi: 10.1016/j.micres.2023.127595 38218095

[12] Wang L Bierbrier R Drucker AM Chan AW . Noncutaneous and cutaneous cancer risk in patients with atopic dermatitis: A systematic review and meta-analysis. JAMA Dermatol. (2020) 156:158–71. doi: 10.1001/jamadermatol.2019.3786 PMC6990857 31825457

[13] Wang H Diepgen TL . Atopic dermatitis and cancer risk. Br J Dermatol. (2006) 154:205–10. doi: 10.1111/j.1365-2133.2005.07077.x 16433786

[14] Hasan I Parsons L Duran S Zinn Z . Dupilumab therapy for atopic dermatitis is associated with increased risk of cutaneous T cell lymphoma: A retrospective cohort study. J Am Acad Dermatol. (2024) 91:255–8. doi: 10.1016/j.jaad.2024.03.039 38588818

[15] Luo J Craver A Bahl K Stepniak L Moore K King J . Etiology of non-hodgkin lymphoma: A review from epidemiologic studies. J Natl Cancer Cent. (2022) 2:226–34. doi: 10.1016/j.jncc.2022.08.003 PMC11256700 39036553

[16] Bispo JAB Pinheiro PS Kobetz EK . Epidemiology and etiology of leukemia and lymphoma. Cold Spring Harb Perspect Med. (2020) 10(6):a034819. doi: 10.1101/cshperspect.a034819 31727680 PMC7263093

[17] Kwatra SG Misery L Clibborn C Steinhoff M . Molecular and cellular mechanisms of itch and pain in atopic dermatitis and implications for novel therapeutics. Clin Transl Immunol. (2022) 11:e1390. doi: 10.1002/cti2.1390 PMC9082890 35582626

[18] Powers CM Piontkowski AJ Orloff J Pulsinelli J Uddin FB Correa Da Rosa J . Risk of lymphoma in patients with atopic dermatitis: A case-control study in the all of us database. J Am Acad Dermatol. (2024) 91:344–6. doi: 10.1016/j.jaad.2024.03.038 38582238

[19] Wan J Shin DB Syed MN Abuabara K Lemeshow AR Fuxench ZCC . Malignancy risk in patients with atopic dermatitis: A population-based cohort study. Br J Dermatol. (2023) 189:53–61. doi: 10.1093/bjd/ljad072 37418646

[20] Rafiq M Hayward A Warren-Gash C Denaxas S Gonzalez-Izquierdo A Lyratzopoulos G . Allergic disease, corticosteroid use, and risk of hodgkin lymphoma: A United Kingdom nationwide case-control study. J Allergy Clin Immunol. (2020) 145:868–76. doi: 10.1016/j.jaci.2019.10.033 PMC7057259 31730878

[21] Castellsague J Kuiper JG Pottegård A Anveden Berglind I Dedman D Gutierrez L . A cohort study on the risk of lymphoma and skin cancer in users of topical tacrolimus, pimecrolimus, and corticosteroids (Joint european longitudinal lymphoma and skin cancer evaluation - joelle study). Clin Epidemiol. (2018) 10:299–310. doi: 10.2147/clep.S146442 29559812 PMC5856050

[22] Matthewman J Schultze A Strongman H Bhaskaran K Roberts A Denaxas S . Cohort studies on 71 outcomes among people with atopic eczema in uk primary care data. Nat Commun. (2024) 15:9573. doi: 10.1038/s41467-024-54035-1 39505873 PMC11541564

[23] Söderberg KC Hagmar L Schwartzbaum J Feychting M . Allergic conditions and risk of hematological Malignancies in adults: A cohort study. BMC Public Health. (2004) 4:51. doi: 10.1186/1471-2458-4-51 15527506 PMC534807

[24] D’Arcy M Rivera DR Grothen A Engels EA . Allergies and the subsequent risk of cancer among elderly adults in the United States. Cancer Epidemiol Biomarkers Prev. (2019) 28:741–50. doi: 10.1158/1055-9965.Epi-18-0887 PMC6449198 30700443

[25] Liu Q Chen L Wang Y Wang X Lewis SJ Wang J . Atopic dermatitis and risk of 14 site-specific cancers: A mendelian randomization study. J Eur Acad Dermatol Venereol. (2023) 37:2490–7. doi: 10.1111/jdv.19380 37478287

[26] Callen JP Bernardi DM Clark RA Weber DA . Adult-onset recalcitrant eczema: A marker of noncutaneous lymphoma or leukemia. J Am Acad Dermatol. (2000) 43:207–10. doi: 10.1067/mjd.2000.105502 10906639

[27] Serra-García L Riera-Monroig J Riquelme-Mc-Loughlin C Morgado-Carrasco-Daniel C . Chronic prurigo as a onset of hodgkin’s lymphoma. Med Clin (Barc). (2021) 156:47. doi: 10.1016/j.medcli.2019.10.004 31753320

[28] Moher D Liberati A Tetzlaff J Altman DG . Preferred reporting items for systematic reviews and meta-analyses: the prisma statement. Ann Intern Med. (2009) 151:264–9,w64. doi: 10.7326/0003-4819-151-4-200908180-00135 19622511

[29] Tan NKW Tang A MacAlevey N Tan BKJ Oon HH . Risk of suicide and psychiatric disorders among isotretinoin users: A meta-analysis. JAMA Dermatol. (2024) 160:54–62. doi: 10.1001/jamadermatol.2023.4579 38019562 PMC10687715

[30] Higgins J Green S . Tools for assessing methodological quality or risk of bias in non-randomized studies. In: Cochrane Handbook for systematic reviews of interventions version, vol. 5. (2011) London, UK: The Cochrane Collaboration.

[31] Jayedi A Soltani S Motlagh SZ Emadi A Shahinfar H Moosavi H . Anthropometric and adiposity indicators and risk of type 2 diabetes: systematic review and dose-response meta-analysis of cohort studies. Bmj. (2022) 376:e067516. doi: 10.1136/bmj-2021-067516 35042741 PMC8764578

[32] Garcia L Pearce M Abbas A Mok A Strain T Ali S . Non-occupational physical activity and risk of cardiovascular disease, cancer and mortality outcomes: A dose-response meta-analysis of large prospective studies. Br J Sports Med. (2023) 57:979–89. doi: 10.1136/bjsports-2022-105669 PMC10423495 36854652

[33] Su L Yang ZT Qu H Luo CL Yuan GX Wu J . Effect of antioxidants supplementation on erectile dysfunction: A systematic review and meta-analysis of randomized controlled trials. Sex Med Rev. (2022) 10:754–63. doi: 10.1016/j.sxmr.2022.01.002 35246405

[34] Arellano FM Wentworth CE Arana A Fernández C Paul CF . Risk of lymphoma following exposure to calcineurin inhibitors and topical steroids in patients with atopic dermatitis. J Invest Dermatol. (2007) 127:808–16. doi: 10.1038/sj.jid.5700622 17096020

[35] Arellano FM Arana A Wentworth CE Fernández-Vidaurre C Schlienger RG Conde E . Lymphoma among Patients with Atopic Dermatitis and/or Treated with Topical Immunosuppressants in the United Kingdom. J Allergy Clin Immunol. (2009) 123:1111–6,116.e1-13. doi: 10.1016/j.jaci.2009.02.028 19361841

[36] Engels EA Parsons R Besson C Morton LM Enewold L Ricker W . Comprehensive evaluation of medical conditions associated with risk of non-hodgkin lymphoma using medicare claims (“Medwas”). Cancer Epidemiol Biomarkers Prev. (2016) 25:1105–13. doi: 10.1158/1055-9965.Epi-16-0212 PMC4930732 27197296

[37] Joshi TP Black TA Fernandez B Friske S Stafford H Strouphauer E . Comorbidities associated with mycosis fungoides: A case-control study in the all of us database. J Am Acad Dermatol. (2023) 88:686–8. doi: 10.1016/j.jaad.2022.07.003 35817334

[38] Jung SW Lee S . All-cause and cause-specific mortality risk associated with atopic dermatitis: A korean nationwide population-based study. J Eur Acad Dermatol Venereol. (2023) 37:e618–e20. doi: 10.1111/jdv.18803 36463419

[39] Kaul S Belzberg M Hughes JM Mahadevan V Khanna R Bakhshi PR . Comorbidities in mycosis fungoides and racial differences in co-existent lymphomatoid papulosis: A cross-sectional study of 580 patients in an urban tertiary care center. Medicines (Basel). (2019) 7(1):1. doi: 10.3390/medicines7010001 31888015 PMC7168128

[40] Mansfield KE Schmidt SAJ Darvalics B Mulick A Abuabara K Wong AYS . Association between atopic eczema and cancer in England and Denmark. JAMA Dermatol. (2020) 156:1086–97. doi: 10.1001/jamadermatol.2020.1948 PMC7315391 32579178

[41] Morales MM Olsen J Johansen P Kaerlev L Guénel P Arveux P . Viral infection, atopy and mycosis fungoides: A European multicentre case-control study. Eur J Cancer. (2003) 39:511–6. doi: 10.1016/s0959-8049(02)00773-6 12751383

[42] Pierog O Bao A Rozati S . The association of atopic dermatitis and cutaneous T-cell lymphoma: A multicentre cohort study. J Eur Acad Dermatol Venereol. (2025) (2025) 39(2):e172-3. doi: 10.1111/jdv.20243 38994887

[43] Ruff S Egeberg A Andersen YMF Gislason G Skov L Thyssen JP . Prevalence of cancer in adult patients with atopic dermatitis: A nationwide study. Acta Derm Venereol. (2017) 97:1127–9. doi: 10.2340/00015555-2703 28512668

[44] Tuyp E Burgoyne A Aitchison T MacKie R . A case-control study of possible causative factors in mycosis fungoides. Arch Dermatol. (1987) 123:196–200. doi: 10.1001/archderm.1987.01660260066015 3813592

[45] Lange-Vejlsgaard G Ralfkiaer E Larsen JK O’Connor N Thomsen K . Fatal cutaneous T cell lymphoma in a child with atopic dermatitis. J Am Acad Dermatol. (1989) 20:954–8. doi: 10.1016/s0190-9622(89)70118-3 2785543

[46] Dobos G de Masson A Ram-Wolff C Beylot-Barry M Pham-Ledard A Ortonne N . Epidemiological changes in cutaneous lymphomas: an analysis of 8593 patients from the french cutaneous lymphoma registry. Br J Dermatol. (2021) 184:1059–67. doi: 10.1111/bjd.19644 33131055

[47] Hristov AC Tejasvi T Wilcox RA . Mycosis fungoides and sézary syndrome: 2019 update on diagnosis, risk-stratification, and management. Am J Hematol. (2019) 94:1027–41. doi: 10.1002/ajh.25577 31313347

[48] Miyashiro D Vivarelli AG Gonçalves F Cury-Martins J Sanches JA . Progression of mycosis fungoides after treatment with dupilumab: A case report. Dermatol Ther. (2020) 33:e13880. doi: 10.1111/dth.13880 32558148

[49] Chiba T Nagai T Osada SI Manabe M . Diagnosis of mycosis fungoides following administration of dupilumab for misdiagnosed atopic dermatitis. Acta Derm Venereol. (2019) 99:818–9. doi: 10.2340/00015555-3208 31045233

[50] Schürch CM Federmann B Quintanilla-Martinez L Fend F . Tumor heterogeneity in lymphomas: A different breed. Pathobiology. (2018) 85:130–45. doi: 10.1159/000475530 28719907

[51] Swerdlow SH Campo E Pileri SA Harris NL Stein H Siebert R . The 2016 revision of the world health organization classification of lymphoid neoplasms. Blood. (2016) 127:2375–90. doi: 10.1182/blood-2016-01-643569 PMC4874220 26980727

[52] Bowzyk-Al-Naeeb A Ajithkumar T Behan S Hodson DJ . Non-hodgkin lymphoma. Bmj. (2018) 362:k3204. doi: 10.1136/bmj.k3204 30135071

[53] Lyapichev KA You MJ . Unusual presentation of classic hodgkin lymphoma. Blood. (2019) 133:502. doi: 10.1182/blood-2018-10-878058 30705049

[54] Esteves M Nogueira A Azevedo F . Advanced hodgkin lymphoma with extensive cutaneous infiltration. Indian J Dermatol Venereol Leprol. (2022) 88:680–1. doi: 10.25259/ijdvl_1071_19 33666052

[55] Legendre L Barnetche T Mazereeuw-Hautier J Meyer N Murrell D Paul C . Risk of lymphoma in patients with atopic dermatitis and the role of topical treatment: A systematic review and meta-analysis. J Am Acad Dermatol. (2015) 72:992–1002. doi: 10.1016/j.jaad.2015.02.1116 25840730

[56] Silverberg JI Thyssen JP Paller AS Drucker AM Wollenberg A Lee KH . What’s in a name? Atopic dermatitis or atopic eczema, but not eczema alone. Allergy. (2017) 72:2026–30. doi: 10.1111/all.13225 28605026

[57] Gumina ME Hooper MJ Zhou XA Koralov SB . Role of antigenic stimulation in cutaneous T-cell lymphomas. J Invest Dermatol. (2024) 144:755–63. doi: 10.1016/j.jid.2023.10.023 PMC10960716 38149950

[58] Nocturne G Virone A Ng WF Le Guern V Hachulla E Cornec D . Rheumatoid factor and disease activity are independent predictors of lymphoma in primary sjögren’s syndrome. Arthritis Rheumatol. (2016) 68:977–85. doi: 10.1002/art.39518 26606524

[59] Margolis DJ . Atopic dermatitis: filaggrin and skin barrier dysfunction. Br J Dermatol. (2022) 186:396. doi: 10.1111/bjd.20946 35128630

[60] Geoghegan JA Irvine AD Foster TJ . Staphylococcus aureus and atopic dermatitis: A complex and evolving relationship. Trends Microbiol. (2018) 26:484–97. doi: 10.1016/j.tim.2017.11.008 29233606

[61] Patrick GJ Archer NK Miller LS . Which way do we go? Complex interactions in atopic dermatitis pathogenesis. J Invest Dermatol. (2021) 141:274–84. doi: 10.1016/j.jid.2020.07.006 PMC7855294 32943210

[62] Krejsgaard T Lindahl LM Mongan NP Wasik MA Litvinov IV Iversen L . Malignant inflammation in cutaneous T-cell lymphoma-a hostile takeover. Semin Immunopathol. (2017) 39:269–82. doi: 10.1007/s00281-016-0594-9 PMC5368200 27717961

[63] Willerslev-Olsen A Krejsgaard T Lindahl LM Litvinov IV Fredholm S Petersen DL . Staphylococcal enterotoxin a (Sea) stimulates stat3 activation and il-17 expression in cutaneous T-cell lymphoma. Blood. (2016) 127:1287–96. doi: 10.1182/blood-2015-08-662353 PMC4786838 26738536

[64] Fanok MH Sun A Fogli LK Narendran V Eckstein M Kannan K . Role of dysregulated cytokine signaling and bacterial triggers in the pathogenesis of cutaneous T-cell lymphoma. J Invest Dermatol. (2018) 138:1116–25. doi: 10.1016/j.jid.2017.10.028 PMC5912980 29128259

[65] Milner JD . Primary atopic disorders. Annu Rev Immunol. (2020) 38:785–808. doi: 10.1146/annurev-immunol-042718-041553 32126183

[66] Human germline gain-of-function in stat6: from severe allergic disease to lymphoma and beyond. Trends Immunol. (2024) 45:138–53. doi: 10.1016/j.it.2023.12.003 38238227

[67] Sharma M Leung D Momenilandi M Jones LCW Pacillo L James AE . Human germline heterozygous gain-of-function stat6 variants cause severe allergic disease. J Exp Med. (2023) 220(5):e20221755. doi: 10.1084/jem.20221755 36884218 PMC10037107

[68] Suratannon N Ittiwut C Dik WA Ittiwut R Meesilpavikkai K Israsena N . A germline stat6 gain-of-function variant is associated with early-onset allergies. J Allergy Clin Immunol. (2023) 151:565–71.e9. doi: 10.1016/j.jaci.2022.09.028 36216080

[69] Yildiz M Li H Bernard D Amin NA Ouillette P Jones S . Activating stat6 mutations in follicular lymphoma. Blood. (2015) 125:668–79. doi: 10.1182/blood-2014-06-582650 PMC4729538 25428220

[70] Tiacci E Ladewig E Schiavoni G Penson A Fortini E Pettirossi V . Pervasive mutations of jak-stat pathway genes in classical hodgkin lymphoma. Blood. (2018) 131:2454–65. doi: 10.1182/blood-2017-11-814913 PMC6634958 29650799

[71] Mentz M Keay W Strobl CD Antoniolli M Adolph L Heide M . Parp14 is a novel target in stat6 mutant follicular lymphoma. Leukemia. (2022) 36:2281–92. doi: 10.1038/s41375-022-01641-x PMC9417990 35851155

[72] Freitas E Gooderham M Torres T . New topical therapies in development for atopic dermatitis. Drugs. (2022) 82:843–53. doi: 10.1007/s40265-022-01722-2 35596877

[73] Pena J Zameza PA Pixley JN Remitz A Feldman SR . A comparison of topical corticosteroids and topical calcineurin inhibitors for the treatment of atopic dermatitis. J Allergy Clin Immunol Pract. (2023) 11:1347–59. doi: 10.1016/j.jaip.2023.03.022 36997119

[74] Evens AM Antillón M Aschebrook-Kilfoy B Chiu BC . Racial disparities in hodgkin’s lymphoma: A comprehensive population-based analysis. Ann Oncol. (2012) 23:2128–37. doi: 10.1093/annonc/mdr578 22241896

[75] Cai ZR Chen ML Weinstock MA Kim YH Novoa RA Linos E . Incidence trends of primary cutaneous T-cell lymphoma in the us from 2000 to 2018: A seer population data analysis. JAMA Oncol. (2022) 8:1690–2. doi: 10.1001/jamaoncol.2022.3236 PMC9437821 36048455

[76] Berndt SI Vijai J Benavente Y Camp NJ Nieters A Wang Z . Distinct germline genetic susceptibility profiles identified for common non-hodgkin lymphoma subtypes. Leukemia. (2022) 36:2835–44. doi: 10.1038/s41375-022-01711-0 PMC10337695 36273105

