# Supplementary material for: Atopic dermatitis and lymphoma risk: a systematic review and meta-analysis
Source: Front Oncol. 2025 Apr 14;15:1580550. doi: 10.3389/fonc.2025.1580550 (PMC12034737; doi:10.3389/fonc.2025.1580550)
Supplement: Supplementary file 1 [file DataSheet1.docx]

**Atopic dermatitis and lymphoma risk: a systematic review and meta-analysis**

**“Online Supplementary Material”**

Supplemental Appendix 1:

Full search strategy (11th August 2024): PubMed

| **Searches** |
| --- |
| #1 "dermatitis, atopic"[MeSH Terms] OR "atopic dermatitis"[Title/Abstract] OR "eczema"[Title/Abstract]  #2 "Lymphoma"[MeSH Terms] OR "lymphoma"[Title/Abstract] OR "lymphomas"[Title/Abstract] OR "lymphoproliferative neoplasms"[Title/Abstract] OR "chronic lymphatic leukemia"[Title/Abstract]  #3 #1 AND #2  #4 "animals"[MeSH Terms] NOT "humans"[MeSH Terms]  #5 #3 NOT #4  #6 limit #5 to English language |

Supplemental Appendix 2: Full-text publications excluded with reason

| **Online Supplementary Reference** | **Reason for exclusion** |
| --- | --- |
| (1-24) | Inappropriate study population (n=24) |
| (25-35) | Inappropriate study type (n=11) |
| (36-42) | Inappropriate outcome (n=7) |
| (43) | Overlapping population (n=1) |

**Reference**

1. Aschebrook-Kilfoy B, Cocco P, La Vecchia C, Chang ET, Vajdic CM, Kadin ME, et al. Medical history, lifestyle, family history, and occupational risk factors for mycosis fungoides and Sézary syndrome: the InterLymph Non-Hodgkin Lymphoma Subtypes Project. Journal of the National Cancer Institute Monographs. 2014;2014(48):98-105.

2. Bernstein L, Ross RK. Prior medication use and health history as risk factors for non-Hodgkin's lymphoma: preliminary results from a case-control study in Los Angeles County. Cancer Res. 1992;52(19 Suppl):5510s-5s.

3. Bonaventure A, Orsi L, Rudant J, Goujon-Bellec S, Leverger G, Baruchel A, et al. Genetic polymorphisms of Th2 interleukins, history of asthma or eczema and childhood acute lymphoid leukaemia: findings from the ESCALE study (SFCE). Cancer epidemiology. 2018;55:96‐103.

4. Cerhan JR, Fredericksen ZS, Wang AH, Habermann TM, Kay NE, Macon WR, et al. Design and validity of a clinic-based case-control study on the molecular epidemiology of lymphoma. International journal of molecular epidemiology and genetics. 2011;2(2):95-113.

5. Cozen W, Cerhan JR, Martinez-Maza O, Ward MH, Linet M, Colt JS, et al. The effect of atopy, childhood crowding, and other immune-related factors on non-Hodgkin lymphoma risk. Cancer causes & control : CCC. 2007;18(8):821-31.

6. D'Arcy M, Rivera DR, Grothen A, Engels EA. Allergies and the Subsequent Risk of Cancer among Elderly Adults in the United States. Cancer epidemiology, biomarkers & prevention : a publication of the American Association for Cancer Research, cosponsored by the American Society of Preventive Oncology. 2019;28(4):741-50.

7. Doody MM, Linet MS, Glass AG, Friedman GD, Pottern LM, Boice JD, Jr., et al. Leukemia, lymphoma, and multiple myeloma following selected medical conditions. Cancer causes & control : CCC. 1992;3(5):449-56.

8. Ellison-Loschmann L, Benavente Y, Douwes J, Buendia E, Font R, Alvaro T, et al. Immunoglobulin E levels and risk of lymphoma in a case-control study in Spain. Cancer epidemiology, biomarkers & prevention : a publication of the American Association for Cancer Research, cosponsored by the American Society of Preventive Oncology. 2007;16(7):1492-8.

9. El-Zein M, Parent ME, Kâ K, Siemiatycki J, St-Pierre Y, Rousseau MC. History of asthma or eczema and cancer risk among men: a population-based case-control study in Montreal, Quebec, Canada. Annals of Allergy, Asthma and Immunology. 2010;104(5):378-84.

10. Eriksson NE, Mikoczy Z, Hagmar L. Cancer incidence in 13811 patients skin tested for allergy. Journal of investigational allergology & clinical immunology. 2005;15(3):161-6.

11. Grulich AE, Vajdic CM, Kaldor JM, Hughes AM, Kricker A, Fritschi L, et al. Birth order, atopy, and risk of non-Hodgkin lymphoma. Journal of the National Cancer Institute. 2005;97(8):587-94.

12. Hopkins AM, Lerner K, Grinich E, Ahn J, Choi Y, Hanifin J, et al. The Frequency and Utility of Drug Cessation Trials in Older Adults With Chronic Eczematous Dermatitis of Unknown Etiology: A Retrospective Cohort Study. Dermatitis : contact, atopic, occupational, drug. 2023;34(5):419-24.

13. Huang HH, Shen D, Chan TC, Cho YT, Tang CH, Chu CY. Association Between the Use of Topical Calcineurin Inhibitors and the Risk of Cancer Among Patients with Atopic Dermatitis: A Nationwide, Population-Based, Retrospective Cohort Study. Am J Clin Dermatol. 2023;24(5):799-808.

14. Linabery AM, Prizment AE, Anderson KE, Cerhan JR, Poynter JN, Ross JA. Allergic diseases and risk of hematopoietic malignancies in a cohort of postmenopausal women: A report from the Iowa women's health study. Cancer Epidemiology Biomarkers and Prevention. 2014;23(9):1903-12.

15. Mbulaiteye SM, Morton LM, Sampson JN, Chang ET, Costas L, de Sanjosé S, et al. Medical history, lifestyle, family history, and occupational risk factors for sporadic Burkitt lymphoma/leukemia: the Interlymph Non-Hodgkin Lymphoma Subtypes Project. Journal of the National Cancer Institute Monographs. 2014;2014(48):106-14.

16. Melbye M, Smedby KE, Lehtinen T, Rostgaard K, Glimelius B, Munksgaard L, et al. Atopy and risk of non-Hodgkin lymphoma. Journal of the National Cancer Institute. 2007;99(2):158-66.

17. Morton LM, Slager SL, Cerhan JR, Wang SS, Vajdic CM, Skibola CF, et al. Etiologic heterogeneity among non-Hodgkin lymphoma subtypes: the InterLymph Non-Hodgkin Lymphoma Subtypes Project. Journal of the National Cancer Institute Monographs. 2014;2014(48):130-44.

18. Rafiq M, Hayward A, Warren-Gash C, Denaxas S, Gonzalez-Izquierdo A, Lyratzopoulos G, et al. Allergic disease, corticosteroid use, and risk of Hodgkin lymphoma: A United Kingdom nationwide case-control study. J Allergy Clin Immunol. 2020;145(3):868-76.

19. Schneeweiss S, Doherty M, Zhu S, Funch D, Schlienger RG, Fernandez-Vidaurre C, et al. Topical treatments with pimecrolimus, tacrolimus and medium- to high-potency corticosteroids, and risk of lymphoma. Dermatology (Basel, Switzerland). 2009;219(1):7-21.

20. Söderberg KC, Hagmar L, Schwartzbaum J, Feychting M. Allergic conditions and risk of hematological malignancies in adults: a cohort study. BMC public health. 2004;4:51.

21. Tan GF, Kong YL, Tan AS, Tey HL. Causes and features of erythroderma. Annals of the Academy of Medicine, Singapore. 2014;43(8):391-4.

22. Vajdic CM, Falster MO, de Sanjose S, Martínez-Maza O, Becker N, Bracci PM, et al. Atopic disease and risk of non-Hodgkin lymphoma: an InterLymph pooled analysis. Cancer Res. 2009;69(16):6482-9.

23. Vajdic CM, Fritschi L, Grulich AE, Kaldor JM, Benke G, Kricker A, et al. Atopy, exposure to pesticides and risk of non-Hodgkin lymphoma. Int J Cancer. 2007;120(10):2271-4.

24. Zhang Y, Holford TR, Leaderer B, Zahm SH, Boyle P, Morton LM, et al. Prior medical conditions and medication use and risk of non-Hodgkin lymphoma in Connecticut United States women. Cancer causes & control : CCC. 2004;15(4):419-28.

25. Arnold JD, Crockett RM, Kirkorian AY. Hospital readmissions among patients with skin disease: A retrospective cohort study. Journal of the American Academy of Dermatology. 2018;79(4):696-701.

26. De Luca DA, Maianski Z, Averbukh M. A study of skin disease spectrum occurring in Angola phototype V–VI population in Luanda. International Journal of Dermatology. 2018;57(7):849-55.

27. Hagströmer L, Ye W, Nyrén O, Emtestam L. Incidence of cancer among patients with atopic dermatitis. Archives of dermatology. 2005;141(9):1123-7.

28. Hedderson MM, Asgari MM, Xu F, Quesenberry CP, Sridhar S, Geier J, et al. Rates of malignancies among patients with moderate to severe atopic dermatitis: A retrospective cohort study. BMJ Open. 2023;13(3).

29. Hui RL, Lide W, Chan J, Schottinger J, Yoshinaga M, Millares M. Association between exposure to topical tacrolimus or pimecrolimus and cancers. The Annals of pharmacotherapy. 2009;43(12):1956-63.

30. Kiiski V, Ukkola-Vuoti L, Vikkula J, Ranta M, Lassenius MI, Kopra J. Effect of Disease Severity on Comorbid Conditions in Atopic Dermatitis: Nationwide Registry-Based Investigation in Finnish Adults. Acta dermato-venereologica. 2023;103:adv00882.

31. Mangold AR, Thompson AK, Davis MD, Saulite I, Cozzio A, Guenova E, et al. Early clinical manifestations of Sézary syndrome: A multicenter retrospective cohort study. J Am Acad Dermatol. 2017;77(4):719-27.

32. Margolis DJ, Abuabara K, Hoffstad OJ, Wan J, Raimondo D, Bilker WB. Association Between Malignancy and Topical Use of Pimecrolimus. JAMA Dermatol. 2015;151(6):594-9.

33. Munzenberger PJ, Montejo JM. Safety of topical calcineurin inhibitors for the treatment of atopic dermatitis. Pharmacotherapy. 2007;27(7):1020-8.

34. Wysocka M, Kossenkov AV, Benoit BM, Troxel AB, Singer E, Schaffer A, et al. CD164 and FCRL3 are highly expressed on CD4+CD26- T cells in Sézary syndrome patients. The Journal of investigative dermatology. 2014;134(1):229-36.

35. Yanik EL, Pfeiffer RM, Freedman DM, Weinstock MA, Cahoon EK, Arron ST, et al. Spectrum of immune-related conditions associated with risk of keratinocyte cancers among elderly adults in the United States. Cancer Epidemiology Biomarkers and Prevention. 2017;26(7):998-1007.

36. Botella-Estrada R, Sanmartin O, Oliver V, Febrer I, Aliaga A. Erythroderma: A clinicopathological study of 56 cases. Archives of dermatology. 1994;130(12):1503-7.

37. Cozen W, Hamilton AS, Zhao P, Salam MT, Deapen DM, Nathwani BN, et al. A protective role for early oral exposures in the etiology of young adult Hodgkin lymphoma. Blood. 2009;114(19):4014-20.

38. Gibson R, Graham S, Lilienfeld A. Epidemiology of diseases in adult males with leukemia. Journal of the National Cancer Institute. 1976;56(5):891-8.

39. Mehrany K, El-Azhary RA, Bouwhuis SA, Pittelkow MR. Cutaneous T-cell lymphoma and atopy: is there an association? The British journal of dermatology. 2003;149(5):1013-7.

40. Roh YS, Huang AH, Sutaria N, Choi U, Wongvibulsin S, Choi J, et al. Real-world comorbidities of atopic dermatitis in the US adult ambulatory population. Journal of the American Academy of Dermatology. 2022;86(4):835-45.

41. Rubenstein M, Duvic M. Cutaneous manifestations of Hodgkin's disease. Int J Dermatol. 2006;45(3):251-6.

42. Wang JH, Derkach A, Pfeiffer RM, Engels EA. Immune-related conditions and cancer-specific mortality among older adults with cancer in the United States. International Journal of Cancer. 2022;151(8):1216-27.

43. Arana A, Wentworth CE, Fernández-Vidaurre C, Schlienger RG, Conde E, Arellano FM. Incidence of cancer in the general population and in patients with or without atopic dermatitis in the U.K. The British journal of dermatology. 2010;163(5):1036-43.


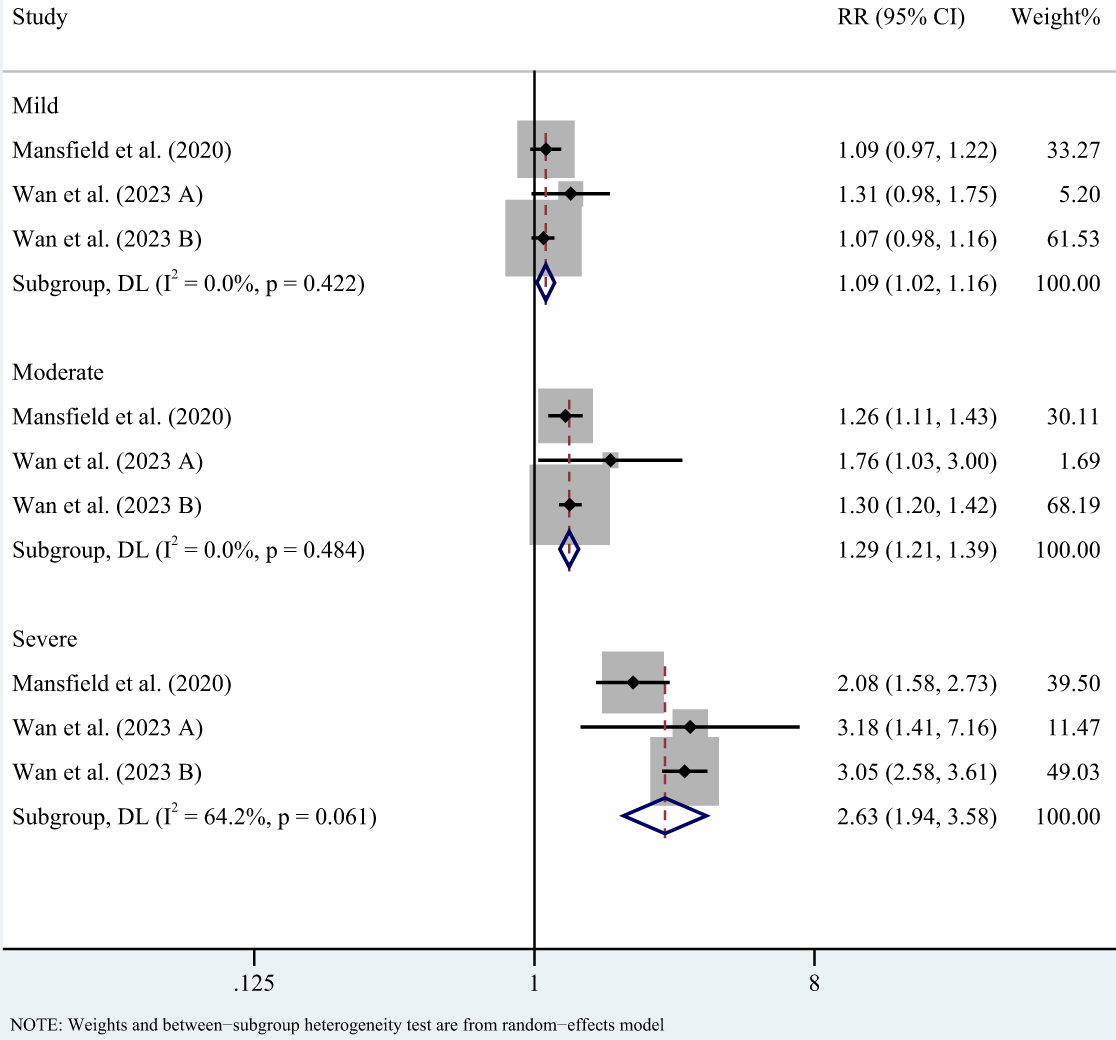


Supplemental Figure 1: Forest plot for subgroup analysis of severity of atopic dermatitis (relative risks). CI: confidence interval; DL: DerSimonian-Laird estimate; I^2^: inconsistency.


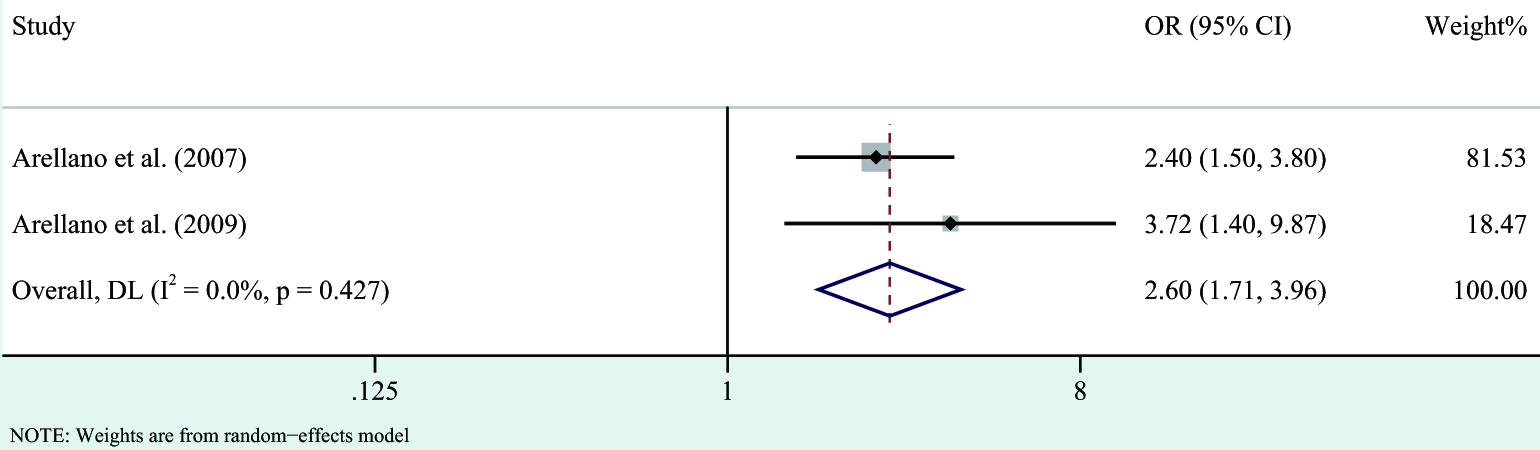


Supplemental Figure 2: Forest plot for subgroup analysis of severity of atopic dermatitis (odds ratios). CI: confidence interval; DL: DerSimonian-Laird estimate; I^2^: inconsistency.


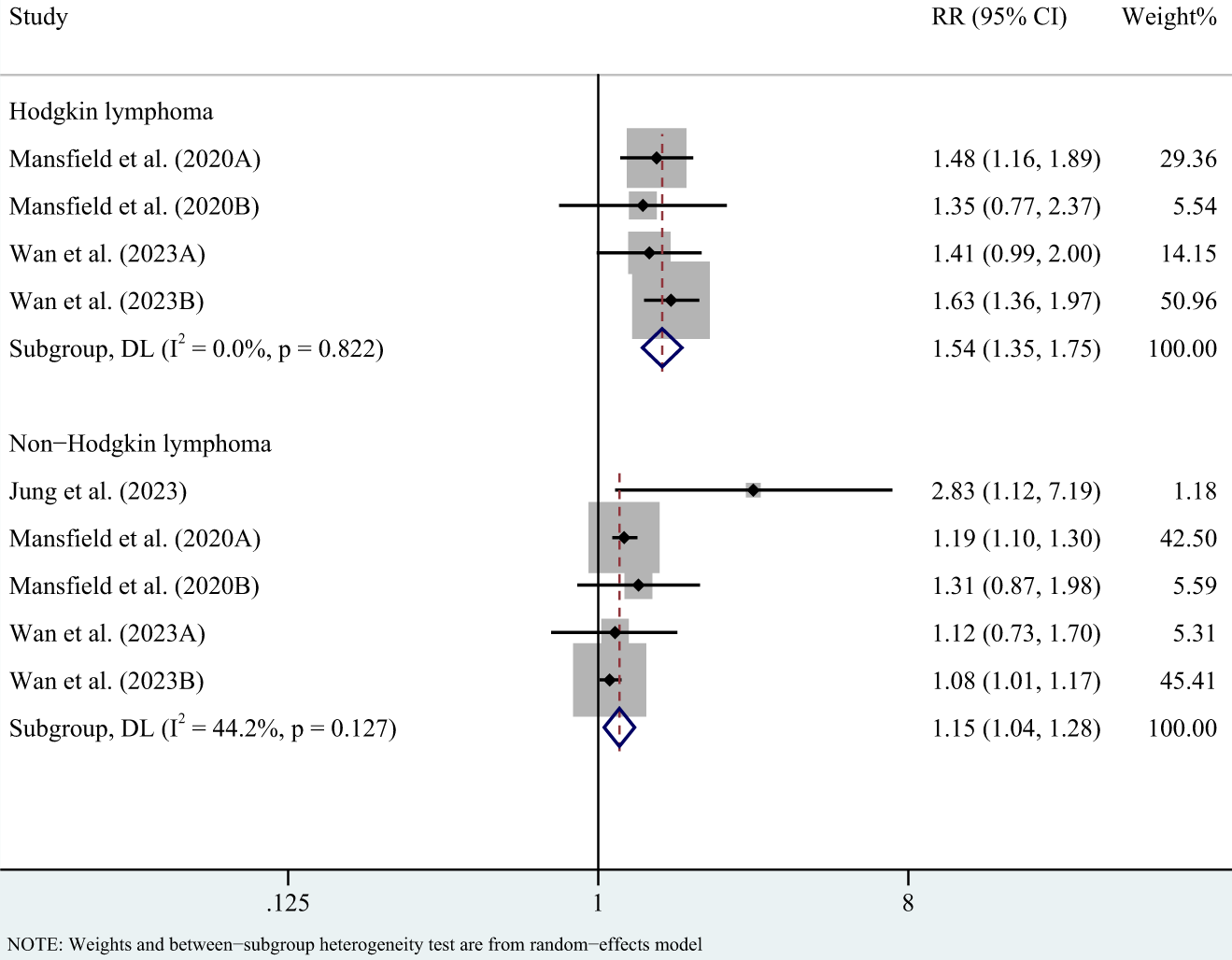


Supplemental Figure 3: Forest plot for subgroup analysis of classification of lymphoma (HL/NHL) (odds ratios). CI: confidence interval; DL: DerSimonian-Laird estimate; I^2^: inconsistency.


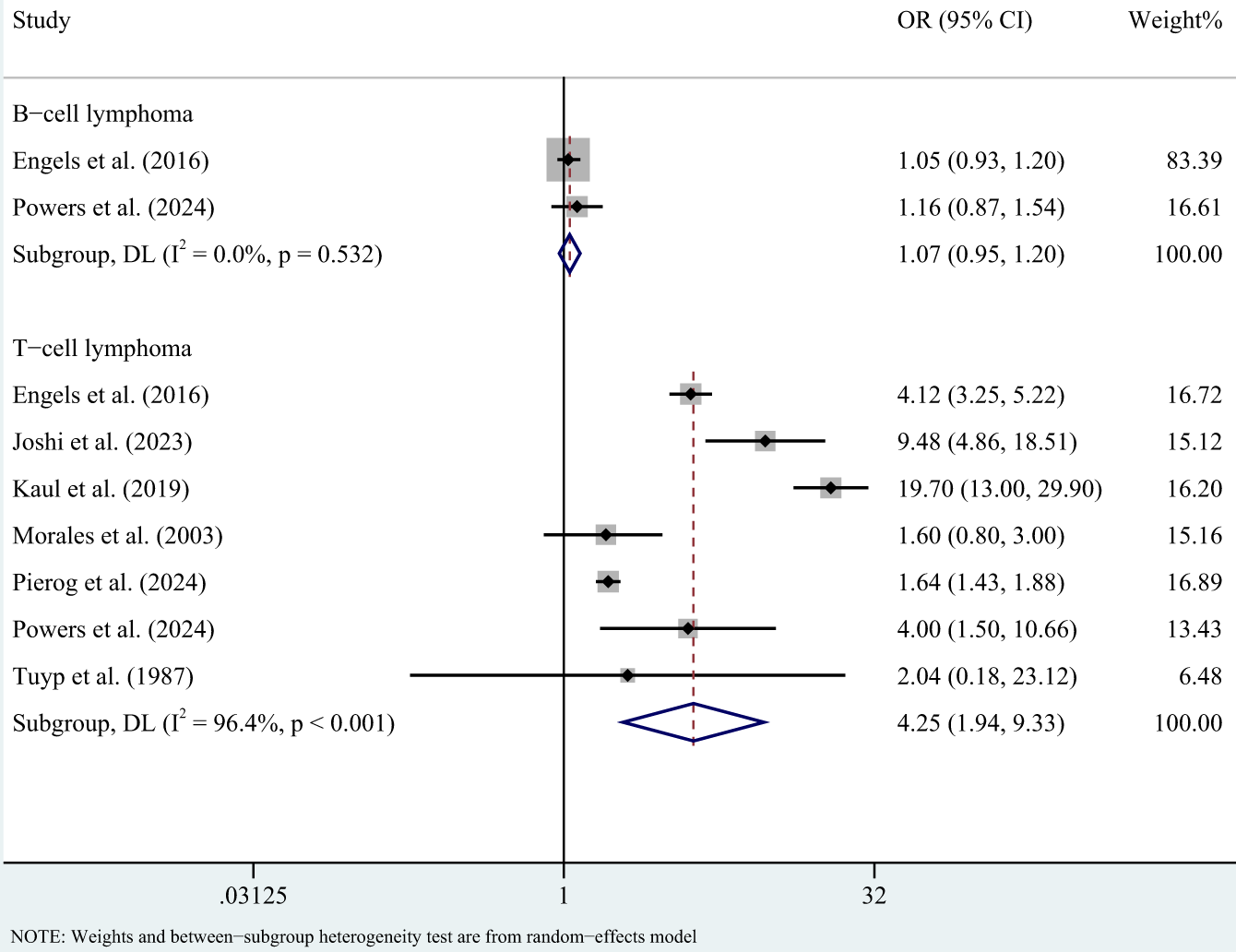


Supplemental Figure 4: Forest plot for subgroup analysis of classification of lymphoma (TCL/BCL) (odds ratios). CI: confidence interval; DL: DerSimonian-Laird estimate; I^2^: inconsistency..


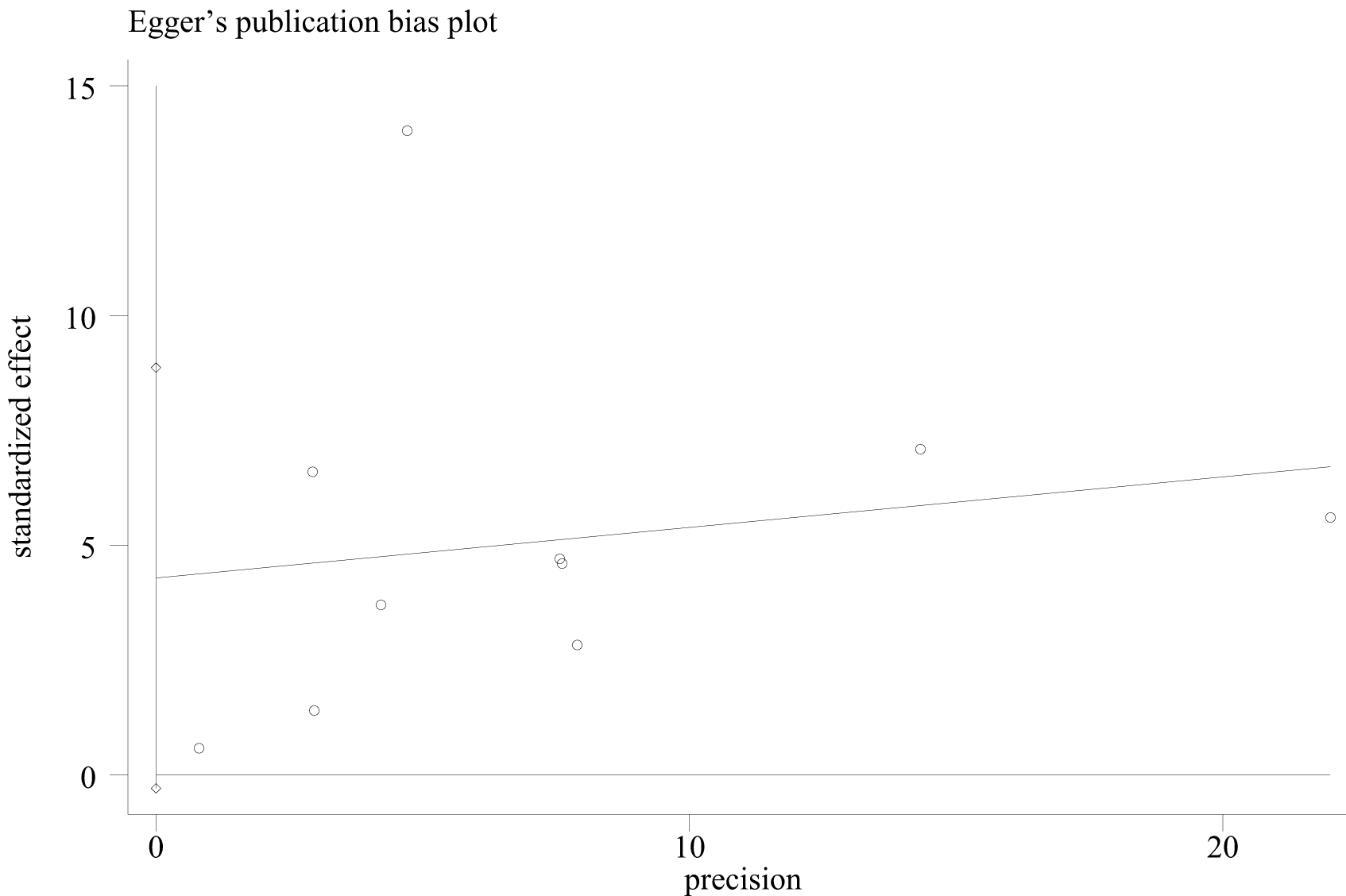


Supplemental Figure 5: The Egger’s test of the associations between AD and the risks of lymphoma in case-control report.


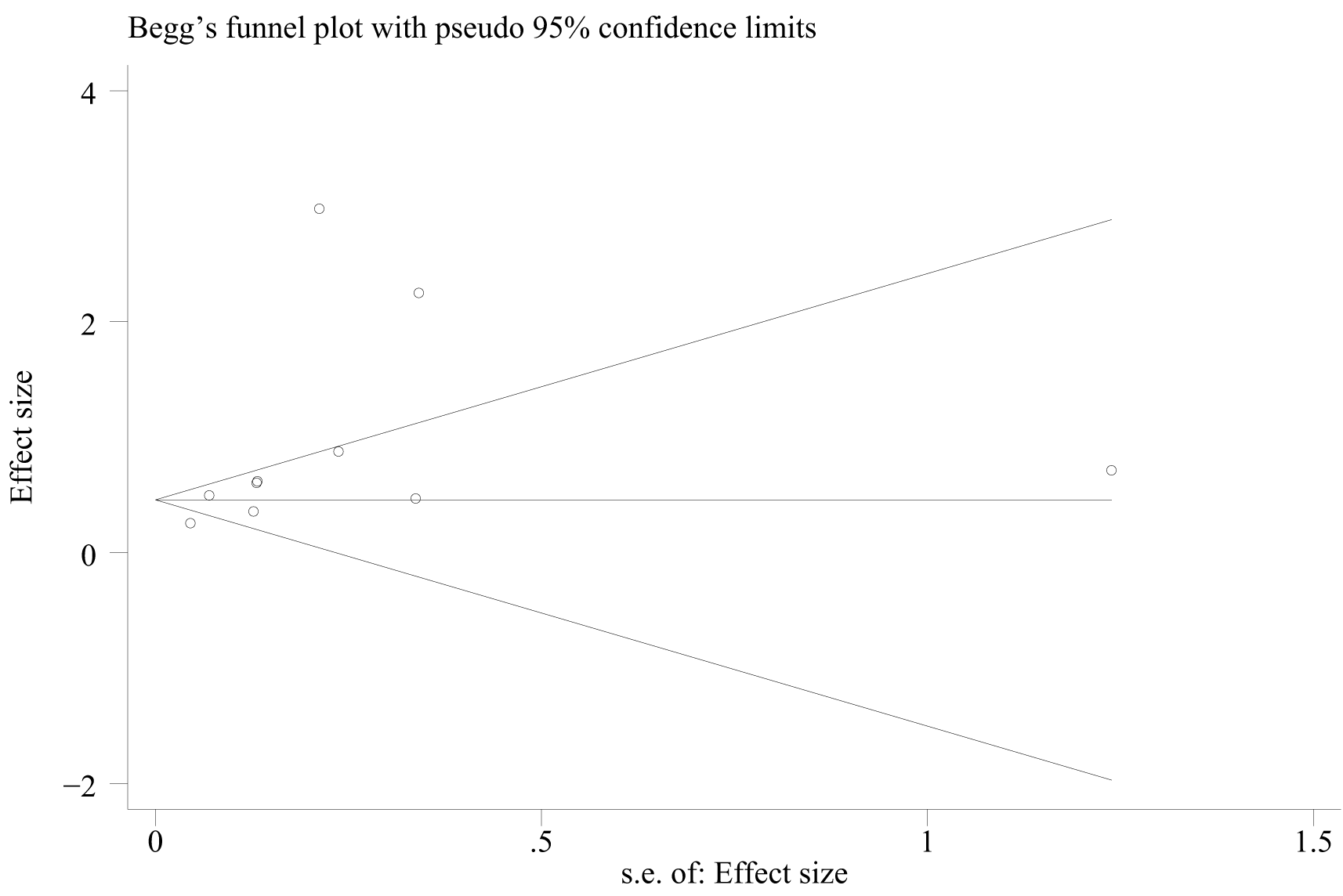


Supplemental Figure 6: The Begg’s test of the associations between AD and the risks of lymphoma in case-control report.
